# Maternal Health Risk Detection: Advancing Midwifery with Artificial Intelligence

**DOI:** 10.3390/healthcare13070833

**Published:** 2025-04-06

**Authors:** Katerina D. Tzimourta, Markos G. Tsipouras, Pantelis Angelidis, Dimitrios G. Tsalikakis, Eirini Orovou

**Affiliations:** 1 Biomedical Technology and Digital Health Laboratory, Department of Electrical and Computer Engineering, University of Western Macedonia, 50100 Kozani, Greece; mtsipouras@uowm.gr (M.G.T.); paggelidis@uowm.gr (P.A.); dtsalikakis@uowm.gr (D.G.T.); eorovou@uowm.gr (E.O.); 2 Department of Midwifery, University of Western Macedonia, 50200 Ptolemaida, Greece

**Keywords:** maternal health risk, midwifery, classification, machine learning, Random Forests, pregnancy care, data-driven healthcare

## Abstract

**Background/Objectives**: Maternal health risks remain one of the critical challenges in the world, contributing much to maternal and infant morbidity and mortality, especially in the most vulnerable populations. In the modern era, with the recent progress in the area of artificial intelligence and machine learning, much promise has emerged with regard to achieving the goal of early risk detection and its management. This research is set out to relate high-risk, low-risk, and mid-risk maternal health using machine learning algorithms based on physiological data. **Materials and Methods**: The applied dataset contains 1014 instances (i.e., cases) with seven attributes (i.e., variables), namely, Age, SystolicBP, DiastolicBP, BS, BodyTemp, HeartRate, and RiskLevel. The preprocessed dataset used was then trained and tested with six classifiers using 10-fold cross-validation. Finally, the performance metrics of the models erre compared using metrics like Accuracy, Precision, and the True Positive Rate. **Results**: The best performance was found for the Random Forest, also reaching the highest values for Accuracy (88.03%), TP Rate (88%), and Precision (88.10%), showing its robustness in handling maternal health risk classification. The mid-risk category was the most challenging across all the models, characterized by lowered Recall and Precision scores, hence underlining class imbalance as one of the bottlenecks in performance. **Conclusions**: Machine learning algorithms hold strong potential for improving maternal health risk prediction. The findings underline the place of machine learning in advancing maternal healthcare by driving more data-driven and personalized approaches.

## 1. Introduction

Maternal health is still one of the most challenging areas of concern on the global platform, with inequity in access to care and the quality of the services as the main factors contributing to the high maternal mortality and morbidity rates. The World Health Organization in a recent report estimated that in 2020, approximately 287,000 women died during and following pregnancy and childbirth, from which 94% were in low- and lower-middle-income countries [1]. Such a long-standing challenge has driven the interest of researchers and healthcare providers in looking at novel ways to help improve maternal health outcomes.

Recent development of AI and ML has brought paradigmatic changes in maternal healthcare. Technology has huge potential in improving Diagnostic Accuracy, personalized care, and unequal access to health. For instance, Mapari et al. [2] identified that AI made the key contribution to complications being detected early, treatment being performed in an individualized manner, and patient monitoring being performed from a distance. Further, Bertini et al. [3], through a review of 31 studies on the application of ML models for prediction of perinatal complications, reported performances of up to 95.7% and 99.7%, respectively, with SVM and XGBoost for neonatal mortality. This evidences the capability of ML in exploiting electronic medical records, medical imaging, and biological markers for predictive modeling.

Further research has iterated these applications: Bruno et al. [4] developed logistic regression models to predict severe maternal morbidity with an AUC of 0.937. Equally, Jhee et al. [5] developed a stochastic gradient boosting method for the early onset of pre-eclampsia with biological markers at a highly cited AUC value of 0.924 and an Accuracy of 97.30%. Also, stretching further into fetal health, deep learning-based convolutional neural networks proposed by Zhao et al. [6] were used for the prediction of fetal acidemia. An excellent AUC of 0.978 and Accuracy of 98.4% were observed in explaining the complex physiologic analytes.

These works, while exciting for the promise of ML in this specialty, outline several hurdles ahead as far as the implementation goes of these research findings in clinical practice. Most of the works are still in the experimental phases of study and will require prospective validation and multi-center trials before integration with clinical workflows. Beyond prediction, there is huge potential for AI and ML to contribute towards equity in maternal health service delivery [7]. For instance, AI-driven, virtual assistant-powered telemedicine interventions can increase outreach to health services in rural and underserved populations, thereby reducing disparities in access and improving outcomes. In other words, ML may have a dual role not only in improving clinical services but also bringing more equity in maternal health services, especially in low-resource settings [1].

### Related Work

Over the past few years, there has been increasing interest in the application of machine learning to maternal and perinatal health, pointing to very promising pathways through which the prediction, prevention, and management of pregnancy complications might be improved [8]. Various ML techniques have been adopted for challenges that range from maternal health risks to pregnancy outcomes and have shown great potential for data-driven approaches within obstetrics and gynecology [7,9].

Several studies have presented the application of machine learning in the prediction of perinatal complications. For example, Bertini et al. (2022) discussed the performance of ML in predicting pre-eclampsia and prematurity conditions, putting great emphasis on how electronic medical records and medical imaging have a great contribution to acieving high Predictive Accuracy [3]. Recently, Khadidos et al. (2024) proposed an ensemble ML framework, which has shown how the integration of several algorithms can provide a very accurate prediction of maternal health risks, focusing on Blood Pressure and glucose levels as critical predictive indicators [9].

Other applications involve the usage of ML for risk management rather than simple prediction. Kopanitsa et al. (2023) [10] described CDSSs for early risk identification in pregnancy, emphasizing the role high-value and interpretable models play within this process of decision-making support. These systems leverage structured and semistructured datasets; therefore, they provide complete support not only to the care of patients but also to health organization management.

Examples of more specific applications of ML to obstetric care include preterm birth prediction. Moreira et al. (2018) [11] developed an SVM-based system suitable for mobile health applications, featuring high Predictive Accuracy. Another good example is presented by Wang et al. (2024) [12], who proposed the use of ML for predicting complications in pregnancies achieved with the help of assisted reproduction techniques. They mentioned that demographic and medical history data need to be integrated in order to improve model results.

In this regard, Raza et al. (2022) [13] came up with another proposal of an ensemble learning-based feature engineering approach in the area of maternal health risk analysis, considering major risk factors such as Systolic and Diastolic Blood Pressure. Their paper underlined the importance of handling class imbalances of the dataset for dependable predictions. Complementing these findings, Rahman et al. (2024) [14] illustrated, with specific tasks, how preprocessing could enhance the performance of the SVM in the classification of health risks and ensure significant improvement in Accuracy.

Cumulatively, these studies indicate the transformative potential of machine learning in maternal and perinatal healthcare [15]. Integration of diverse datasets through state-of-the-art computation aptly provides a path toward more complete, timely, and actionable insights, ultimately for better outcomes of mothers and infants. Better model interpretability, attention to data quality, and multi-center validation studies will be the key determinants in realizing the full potential of these technologies in clinical practice [16,17,18].

This paper proposes a methodology for automated maternal health risk classification based on physiological data. There are three classes in this dataset: low risk, mid risk, and high risk. The proposed system investigates some of the most important variables such as Age, Systolic and Diastolic Blood Pressure, level of Blood Sugar, Body Temperature, and Heart Rate. Those features are used to train six machine learning classifiers. The results obtained show that the Random Forest classifier outperforms the rest, with the highest classification Accuracy of 88.03% using 10-fold cross-validation. This goes a long way in showing how good machine learning models can be at the detection of maternal health risks. 

The rest of the paper is organized as follows: Section 2 describes the dataset and the methodology, including preprocessing techniques and machine learning models applied. Section 3 shows the results of the experiments in terms of the classification performance obtained by the algorithms. Section 4 discusses these results in the related work and underlines the impact of optimization techniques such as SMOTE. Concluding this study, Section 5 outlines some further directions for the improvement of applying machine learning within maternal healthcare.

## 2. Materials and Methods

This section describes the dataset and the methodology followed to develop the proposed model. First, a publicly available dataset is collected, followed by its preprocessing to make it quality-oriented for training. Different machine learning classifiers have been implemented based on findings from the literature review to identify the best operating classifiers for similar tasks. The models have been tested on the dataset, and the performances are compared using certain metrics for evaluation.

**Figure 1:**
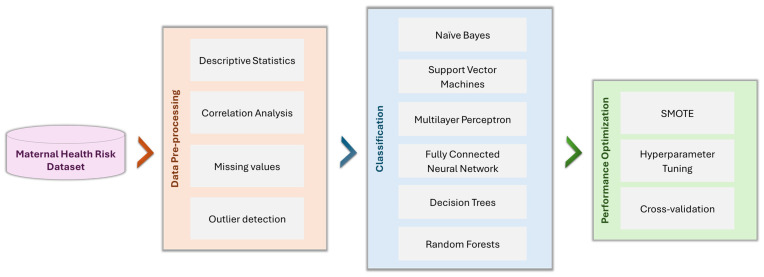
A flowchart of the proposed methodology for maternal health risk classification.

Figure 1 shows the proposed approach for maternal health risk classification. The pipeline starts with the dataset, and data preprocessing is performed through descriptive statistics, analysis of correlation, treatment of missing values, and outlier detection. Further, the Naive Bayes, Support Vector Machines, Multilayer Perceptron, Fully Connected Neural Networks, Decision Trees, and Random Forests are used as classification models. SMOTE, hyperparameter tuning, and cross-validation are among the optimization techniques deployed in order to enhance the performance of the models.

### 2.1. Dataset

In this work, a publicly available dataset was utilized in order to train and test the performance of several classifiers. The data were collected from various hospitals, community clinics, and maternal healthcare centers using an IoT-based risk monitoring system [19].

The dataset includes vital metrics and, in total, 7 significant variables related to maternal mortality. These variables are as follows:

- Age: measured in years;
- Systolic Blood Pressure: measured in mmHg;
- Diastolic Blood Pressure: measured in mmHg;
- Blood Sugar: measured in mmol/L;
- Body Temperature: measured in degrees Fahrenheit;
- Heart Rate: measured in beats per minute;
- Risk Level: a categorical target variable indicating high risk, mid risk, or low risk.

The dataset includes 1014 instances, and all features were used to train and evaluate the machine learning models. The term “instances” is commonly used in the field of machine learning to denote individual data entries or records—in this context, patient cases. Similarly, “features” or “attributes” is a standard term in machine learning, referring to variables used for classification. The distribution of instances across the risk classes is presented in Table 1.

No missing or NaN values were reported in the dataset. The data analysis was performed in the MATLAB environment (R2021a) and the model building and evaluations were conducted using the Waikato WEKA 3.9 Platform. All experiments were run on a personal workstation with an Intel Core i7 processor, 16 GB RAM, and Windows 11 OS.

### 2.2. Preprocessing and Feature Extraction

In order to ensure data quality and consistency, the data were prepared and preprocessed for analysis. To this, descriptive statistics for each variable in the dataset, i.e., Age, Systolic Blood Pressure, Diastolic Blood Pressure, Blood Sugar, Body Temperature, and Heart Raten were extracted and are presented in Table 2. Statistical measures such as minimum, maximum, mean, standard deviation, minimum, percentiles (25th, 50th, 75th), skewness, and kurtosis are included in order to understand the data’s distribution and variability, central tendencies, and spread of each variable. Below, each variable is analyzed.

All features available in the dataset (Age, SystolicBP, DiastolicBP, Blood Sugar, Body Temperature, and Heart Rate) were retained for classification. These are all responsible and significant risk factors for maternal mortality, which is one of the main concerns of the SDG of UN [19,20,21]. Specifically, parameters such as Systolic and Diastolic Blood Pressure, Blood Sugar levels, and Heart Rate are widely recognized as significant maternal health indicators, as they are directly associated with conditions such as pre-eclampsia, gestational hypertension, and gestational diabetes. These conditions are leading causes of maternal and neonatal complications, making their inclusion clinically meaningful. Additionally, Body Temperature can indicate the presence of infections, which also pose significant risks during pregnancy. Initial experiments indicated that removing any of these features led to a decline in overall classification performance. Therefore, no feature elimination was performed, ensuring that the full spectrum of maternal health indicators contributed to model predictions.

#### 2.2.1. Age

In particular, the mean value of the variable Age is 29.87 years, ranging from 10 to 70 years. The standard deviation amounts to 13.47 years, showing that the Age distribution among participants is rather wide. Skewness of 0.783 suggests a mild asymmetry in the data with a left tail containing extreme values. The distribution is a little flatter than the normal bell curve since the kurtosis is −0.391. Most of the Ages range between 19 and 39 years, that is, between the 25th and 75th percentiles. There is only slight skew, with no huge differences in minimum and maximum values in terms of Age outliers.

#### 2.2.2. Systolic Blood Pressure (SystolicBP)

The Mean Systolic Blood Pressure is 113.20 mmHg, and the standard deviation is 18.40 mmHg, which indicates moderate variation. The values range from 70 to 160 mmHg. The skewness is slightly negative (−0.251), which indicates near symmetry, and the kurtosis (−0.613) indicates a flatter distribution than normal. There are no extreme outliers.

#### 2.2.3. Diastolic Blood Pressure (DiastolicBP)

The mean for Diastolic Blood Pressure—76.46 mmHg—is noted, and so is its standard deviation: 13.89 mmHg. Most of the values also fall between 49 and 100 mmHg, mostly within the physiological limits; the skewness of −0.048 and kurtosis of −0.949 also hint at symmetry and the flattened top in distribution. As with the Systolic Blood Pressure reading, no glaring outliers are noted, and that points to standard variability in the heterogeneous sample.

#### 2.2.4. Blood Sugar (BS)

The Blood Sugar variable has a mean value of 11.53 mmol/L and a high standard deviation at 4.19 mmol/L, showing a good deal of variability—one would think with some outliers. The range is between 6 and 19 mmol/L. It has a skewness of 0.348, indicating a slightly asymmetrical distribution, and a kurtosis of −1.244, indicating a flatter distribution than normal.

#### 2.2.5. Body Temperature (BodyTemp)

The Mean Body Temperature is 98.67 °F, with a low standard deviation of 1.37 °F, indicating most values fall in a tight cluster around the mean. All individual values are within the limits of 98 to 103 degrees Fahrenheit—these represent typical variations and hence are consistent with normal bodily variations. Still, the values for skewness and kurtosis indicate right-skewed distributions (1.747 and 1.436, respectively). This might signify fever cases.

#### 2.2.6. Heart Rate

The Mean Heart Rate is 74.30 bpm, and the standard deviation is 8.09 bpm. The lowest value, 7 bpm, is an implausible value and most likely a measurement or device error. The highest Heart Rate is 90 bpm, which is within normal limits. Skewness (−1.044) and kurtosis (8.399) in this case suggest that this distribution is skewed to the left and quite peaked, indicating that there might be some extreme values in the dataset that influence these statistics.

**Figure 2:**
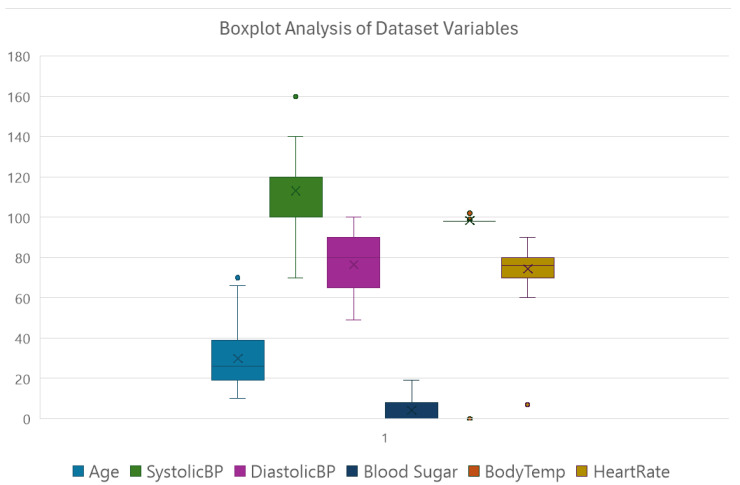
Boxplot analysis of dataset variables.

In summary, based on the analysis, the instance with the least value of Heart Rate, amounting to 7 bpm, was an outlier and hence removed from the dataset. Data are presented in CSV format and thus can easily be integrated into a MATLAB script or the Waikato Weka environment. Among the most important data preprocessing steps, we should also mention the transformation of the target variable, which at first glance is categorical, to a numerical one. That is, the respective conditions of risk, i.e., low-risk, mid-risk, and high-risk, which have been coded as 0, 1, and 2 correspondingly. Above, the overview of data showed that the least value of the Heart Rate variable was 7 bpm, which is not biologically plausible and in all probability was caused by a problem with the imputation. To this effect, two instances with that outlier, initially classified as low-risk, were identified and preprocessed, and their faulty values were replaced by the mode of 70 bpm before further processing and modeling. Detailed boxplot analysis of the variables for more information about the distribution of data is provided in Figure 2.

**Figure 3:**
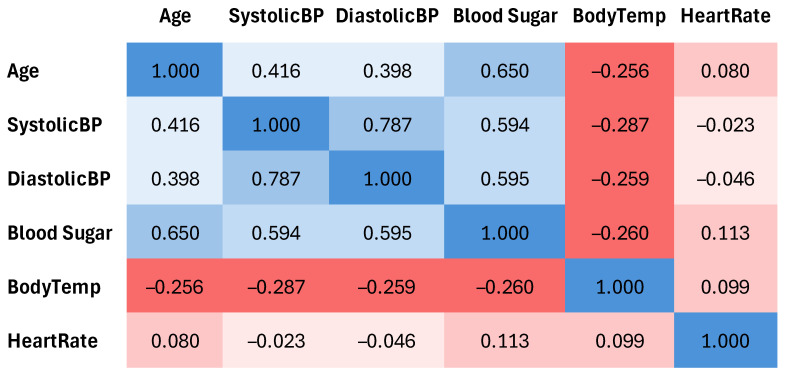
Correlation analysis of features in the dataset. Color intensity represents the strength of the correlation (positive in blue, negative in red), with stronger values indicated by darker shades.

After that, variable correlations were computed and are plotted in Figure 3. Among the given features, Blood Sugar had the highest association with the risk level, and Body Temperature had the lowest. Since the classes for each entry were already labeled in the data, no extra labeling or even transformation of data was required, and the raw data were prepared for application to the proposed analytical framework.

### 2.3. Classification

To achieve the best classification performance, several classifiers were tested, including Naïve Bayes, Support Vector Machines, Multilayer Perceptron, Fully Connected Neural Network (FCNN), Decision Trees, and Random Forests [22].

#### 2.3.1. Naïve Bayes

Naïve Bayes is a simple yet effective probabilistic classifier derived from Bayes’ Theorem, estimating the probability of a particular class given some features. There is, however, an assumption that all the features are conditionally independent—an ideal assumption which can hardly happen in reality; yet, it has proved useful in many practical scenarios. The computational efficiency and capability of handling high-dimensional data make Naïve Bayes very suitable for text applications such as spam filtering and sentiment analysis. Its main weakness is its dependence on the independence assumption, because a poor performance can result whenever this is violated [23].

#### 2.3.2. Support Vector Machines (SVMs)

SVMs are a family of powerful methodologies performing supervised learning, oriented to searching for the best hyperplane to separate data points into classes. In cases when data cannot be separated non-linearly, SVM uses the kernel functions, for instance, polynomial or radial basis functions, to transform the data into higher-dimensional space in which separation may be linearly possible. SVM is versatile and effective, especially with small-sized datasets with complicated boundaries. It has been applied in text classification, bioinformatics, and image recognition. However, there is usually considerable labor involved in fine-tuning parameters and choosing an appropriate kernel for it to perform optimally [24].

#### 2.3.3. Multilayer Perceptron (MLP)

The Multilayer Perceptron is an artificial neural network that applies creditably to intricate and non-linear relationships in data. It has an input layer, one or more hidden layers, and an output layer. In each layer, the neurons are connected by weighted pathways. Training is conducted by backpropagation—a method of modifying these weights to minimize errors. Non-linearity is introduced into the network through activation functions like ReLU and Sigmoid, which allow it to pick up intricacies in the pattern. Their major strengths lie in deep learning areas like image recognition and speech processing. MLPs have a disadvantage: they require a great volume of data and high computational cost. Even so, because of their capability to model complex phenomena, they form indispensable tools in modern machine learning [25].

#### 2.3.4. Fully Connected Neural Network (FCNN)

The FCNN is an elementary structure from which other complex structures for deep learning are derived. It finds its application in modeling multifaceted relationships between different attributes of structured data. The general architecture of FCNNs consists of an input layer, one or more dense hidden layers, and an output layer, where every neuron in one layer is connected to every neuron in the next. These are associated with weights that are updated during training using backpropagation algorithms with the purpose of minimizing errors [24]. Moreover, activation functions such as ReLU and Softmax have been used to provide non-linearity so that the network learns even the most intricate patterns in the data. FCNNs find a wide range of applications in classification and regression problems. Their flexibility in design makes them a very potent weapon for many machine-learning-based applications.

The neural network trained on the Maternal Health Risk Dataset is an FCNN composed of one input layer of 6 attributes, two dense hidden layers, and one output layer. The first hidden layer consists of 8 neurons, the second contains 32 neurons, and both have the ReLU activation function. The output layer contains 3 neurons for the three classes: high risk, low risk, and mid risk, respectively. The Softmax activation function is used for multi-class classification and optimized by categorical cross-entropy loss. A total number of 443 trainable parameters was used, initialized by the Xavier scheme and optimized by the Adam algorithm with a learning rate of 0.001 [26].

#### 2.3.5. Decision Trees

Decision Trees classify data in a recursive manner by splitting them into subsets based on the values of chosen features. Each node of the tree corresponds to the decision rule, while each branch of the tree corresponds to the possible outcome of the rule and ends up with the leaf nodes, which correspond to the class label. Decision Trees are very interpretable and easy to implement; hence, their applications cover financial analysis, customer segmentation, etc. [27].

#### 2.3.6. Random Forests

Ensemble learning methods, such as Random Forests, have a wide applicability for tasks such as classification and regression. During training, the algorithm builds a great number of Decision Trees, after which it sums the results from trees for a better and more robust prediction. Therefore, it uses bagging in broad terms: each tree is trained on a random subset of both data and features. This decreases overfitting and generalizes well. Final prediction is performed by majority voting for classification and averaging for regression. Models of Random Forests allow rather easy interpretation because for any decision, the contribution of every feature can be assessed with the help of some metric, such as feature importance [27].

To optimize model performance, we performed hyperparameter tuning using a grid search strategy for key classifiers:

- Random Forest: The number of trees was tuned within {50, 100, 200}, with the best result at 100 trees.
- SVM: Several different kernel functions were evaluated (linear, RBF, polynomial), with the RBF kernel showing the best performance.
- MLP (Neural Network): The learning rate was tuned within {0.001, 0.01, 0.1}, selecting 0.01 as optimal.
- Decision Tree and Naïve Bayes: Default hyperparameters were used, as preliminary tests showed competitive results without tuning.

## 3. Results

In this work, the 10-fold cross-validation method is used to train and evaluate the six classifiers. The dataset was randomly partitioned into 10 equally sized folds. Each fold served once as the test set, while the remaining nine folds were used for training. This process was repeated 10 times, and the average performance metrics were computed to evaluate model stability and generalizability.

Table 3 presents the performance metrics for each classifier, including the True Positive (TP) Rate (averaged across all classes), Precision (averaged across all classes), and Overall Accuracy. Performance scores ranged from a high of 84.52% by the Random Forest classifier, which also yielded the highest TP Rate of 84.52% and Precision of 84.93%, hence proving it to be superior on this dataset with respect to classification. The Fully Connected Neural Network (FCNN) achieved an Overall Accuracy of 68.93%, comparable to the Multilayer Perceptron (MLP) with 69.03%, and both models displayed moderate Precision and TP Rate scores, indicating their ability to handle complex patterns in the data. The next best in order, after Random Forests, was the Decision Tree classifier, with an Accuracy of 78.60%, followed by SVM, with significantly improved scores of 75.74% Accuracy, 75.70% TP Rate, and 76.30% Precision. Finally, the Naïve Bayes classifier achieved the lowest performance with an Accuracy of 59.07%, Precision of 58.10%, and TP Rate of 59.10%.

In addition to the 10-fold cross-validation approach, we also experimented with an 80–20% split for training and testing algorithms. The results of the 80% training–20% testing split method are tabulated in Table 4. This was achieved by using 80% of the dataset to train the classifiers, while the remaining 20% was held out as the test set for the evaluation of the models.

Table 5 shows the confusion matrix of Random Forests that performed well, which gives a detailed view of the classification performance for each class. The percentage along the main diagonal represents the TP Rate for each class, while off-diagonal elements give the rate at which instances of one class were misclassified as another class. Therefore, 90.07% of instances that fell under the class “high risk” were correctly classified, with the least misclassifications into the classes “low risk” and “mid risk” being 3.31% and 6.62%, respectively. This means a strong power of identification of high-risk instances from among other instances.

The “low risk” class had an Accuracy classification of 81.03%, while its major misclassifications lay in the “mid risk” class, amounting to 16.75%, with only a small proportion, 2.22%, being misclassified as “high risk”. Another performance lay in the “mid-risk” class, which held an Accuracy of 84.23% of its instances classified correctly. However, 10.12% of the “mid-risk” instances were poorly classified into the “low-risk” category, and 5.65% into the “high-risk” class.

Overall, Random Forests performed well in the discrimination between all classes, with their best performances observed in distinguishing “high-risk” from “mid-risk” instances. Given that it is the best model, this model tends to present minor general misclassification, especially between the “low-risk” and “mid-risk” classes. More detailed examination of the confusion matrix emphasizes the dependability of the Random Forest model for matching expert-assigned classifications, where “high-risk” instances are discriminated best at 90.07%.

In an attempt to further improve the performance of the Random Forest classifier, we tried optimization techniques that could potentially help deal with the possible drawbacks in the dataset. More precisely, we applied SMOTE (Synthetic Minority Oversampling Technique) [28] as a way to handle class imbalance since our data showed that the mid-risk category was underrepresented. SMOTE generates synthetic minority class samples by interpolating between existing samples, which helps to balance the dataset, thus allowing the algorithm to recognize the patterns in each of the classes effectively [26]. After the application of SMOTE, the overall Accuracy of the Random Forest model improved substantially to 88.03% from 84.52%, TP Rate 88%, and Precision 88.10%. This proves that SMOTE is effective not only in bias reduction toward the majority classes but also in generalization improvement and the predictive power of the model for all risk levels. Table 6 presents the results after model optimization and Table 7 the updated confusion matrix.

## 4. Discussion

In this work, a machine learning-based approach for the classification of maternal health risk into three categories—high risk, low risk, and mid risk—is presented. The dataset, consisting of 1014 instances and seven attributes (Age, SystolicBP, DiastolicBP, BS, BodyTemp, HeartRate, and RiskLevel), is utilized for the analysis. Preprocessing steps included the normalization of the data and removal of any inconsistencies from the dataset to ensure a good quality. The machine learning classifiers, namely, Random Forests, Fully Connected Neural Networks (FCNNs), Multilayer Perceptron (MLPs), Decision Trees, Support Vector Machines (SVMs), and Naïve Bayes, were then trained and evaluated by taking advantages of 10-fold cross-validation. Classifiers were compared based on the performance metrics such as Accuracy, Precision, and the TP Rate so that the most effective model could be selected for maternal health risk classification.

The outcomes of the current study demonstrated the efficacy of the diverse machine learning algorithms in classifying the maternal health risk. Random Forests performed best among the six classifiers, showing an overall Accuracy of 88.03%, TP Rate of 88.00%, and Precision of 88.10%. Our findings contributes to the currently growing body of literature proving the robustness of ensemble methods in being versatile in handling difficult datasets [27]. The Random Forest efficiently classified three classes of risks, high, low, and mid risks, since it correctly classified most samples with just a small number of misclassifications, as reported by the confusion matrix of this classifier. FCNN and MLP also reached good results, with Overall Accuracies of 68.93% and 69.03%, respectively. FCNN and MLP exhibited a moderate performance that substantiated these neural networks as performing well on complex pattern data. A better approach to hyperparameter tuning and applying data augmentation will contribute much to an improvement in predictive ability. FCNN and MLP showed a good detection for high-risk cases, though they completely failed at detecting mid-risk cases, which is an intrinsic problem present in many real-world classification issues in imbalanced data. Other classic classifiers, such as Decision Trees, Support Vector Machines, and Naïve Bayes, also performed well but could not outperform the Random Forests. The Decision Tree reached an Accuracy of 78.60%; as a model, it benefited from its interpretable and structured decision-making process. Similarly, the Accuracies for SVM and Naïve Bayes were 63.90% and 59.07%, respectively, indicating that these are vulnerable to the complexity and possible multicollinearity at the level of the dataset features.

Confidence intervals were calculated for each classifier’s Accuracy, Precision, and Recall, offering insight into the stability and variation in these metrics (Table 8). For Naive Bayes, the confidence interval for accuracy was approximately 53.61–60.38%, indicating relatively wide variation and suggesting that this classifier may not produce reliable results across different folds. Random Forests, in contrast, achieved both higher mean performances and narrower intervals—for instance, 83.62–87.76% for accuracy—implying a stronger predictive capacity and greater stability. Decision Trees also performed well, though slightly below Random Forests, while Support Vector Machines, MLP, and FCNN exhibited moderate mean values and moderate confidence intervals. These findings illustrate that ensemble-based methods, particularly Random Forests, handle the dataset’s complexity more effectively, yielding consistently better metrics and smaller intervals than other approaches. In practical terms, wide intervals—such as those seen with Naive Bayes—denote considerable performance fluctuation, whereas narrower intervals point to less uncertainty and greater consistency in model outcomes.

Table 9 shows the results of the paired t-test comparing each model’s TP Rate, Precision, and Accuracy against the Random Forests. The metric *p* < 0.001 indicates that these differences are statistically significant, suggesting that each model’s mean TP Rate, Precision, and Accuracy differ substantially and that none of these results are likely due to random chance.

**Figure 4:**
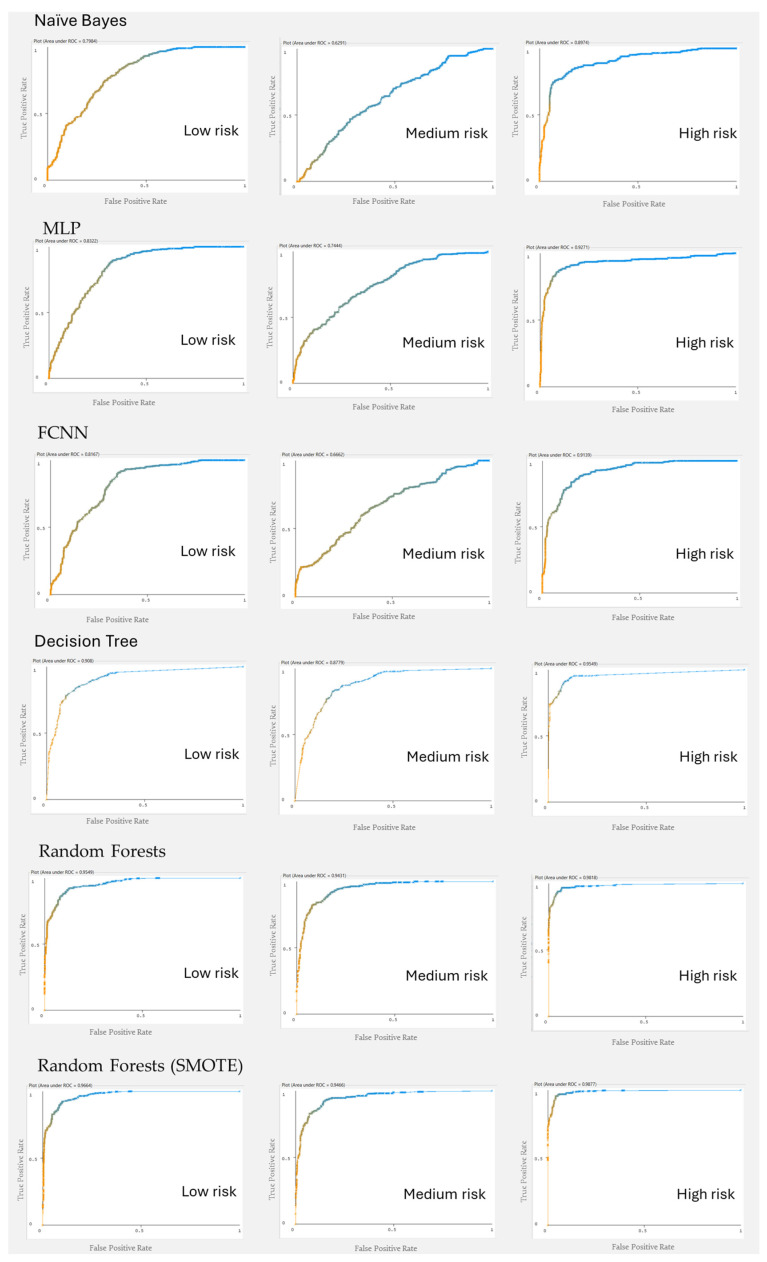
Receiver Operating Characteristic (ROC) curves for different classification models across maternal health risk categories (low risk, medium risk, high risk). Blue lines indicate model performance; orange lines represent random classification (baseline).

To further evaluate model performance across risk levels, Receiver Operating Characteristic (ROC) curves were generated for each classifier and each risk category (low, medium, and high). The ROC curves, presented in Figure 4, illustrate the ability of the models to distinguish between risk levels based on their predicted probabilities.

The high-risk category shows well-defined ROC curves with high Area Under the Curve (AUC) values, particularly for tree-based models like Decision Tree and Random Forest, indicating strong discriminatory power. The low-risk category also exhibits a consistent performance, with most models achieving high True Positive Rates (TPRs) at relatively low False Positive Rates (FPRs). However, as expected, the mid-risk category presents the most classification difficulty, as reflected in the flatter ROC curves and lower AUC scores. This suggests that mid-risk cases overlap significantly with both low- and high-risk groups, making their distinction more challenging. The Random Forest model trained with SMOTE slightly improves classification for mid-risk cases, as seen by the marginal increase in the ROC curve’s steepness, demonstrating the benefits of addressing class imbalance. These visualizations complement the quantitative performance metrics (Accuracy, Precision, Recall) and confirm the earlier findings that mid-risk classification remains a challenge due to the inherent overlap in feature distributions.

The variation in classifier performance justifies the importance of selecting an algorithm with respect to dataset characteristics and application requirements. The class distribution was unequal, with mid-risk samples being the least represented class, and this might be one of the reasons for the poor performance of some classifiers, especially those which are sensitive to class imbalance, such as Naïve Bayes and SVM. Additionally, the steps of preprocessing, including standardization and outlier removal, had a great impact regarding data quality and coherence.

**Figure 5:**
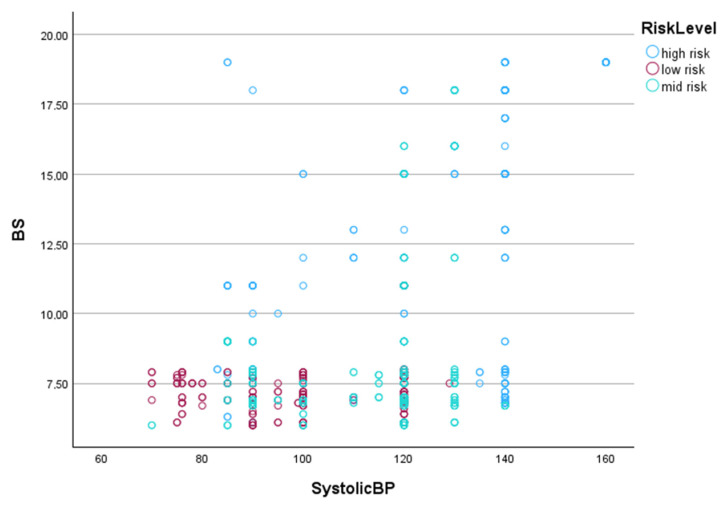
Scatter plot of Systolic Blood Pressure (SystolicBP) vs. Blood Sugar (BS) across maternal health risk levels.

Figure 5 presents a scatter plot illustrating the distribution of SystolicBP and Blood Sugar (BS) values for different maternal health risk categories (low risk, mid risk, and high risk). Each point represents an individual case, with color coding distinguishing between high-risk (blue), low-risk (red), and mid-risk (green) cases. Before generating scatter plots, we performed feature selection using the BestFirst + CfsSubsetEval method in WEKA to determine the most relevant predictors for maternal health risk classification. The analysis identified SystolicBP and Blood Sugar as the two most significant features, consistently selected across multiple validation folds. Based on these results, we visualized these features to examine whether they effectively separate risk categories or exhibit overlapping distributions—particularly for mid-risk cases.

The visualization highlights several key observations regarding the classification challenges of the mid-risk category. First, high-risk cases (blue) tend to have higher Blood Sugar (BS) values and are more dispersed across higher Systolic BP values, particularly above 120 mmHg. This indicates that elevated Blood Sugar levels and hypertension are strong indicators of high-risk cases. Second, low-risk cases (red) cluster more tightly in the lower ranges of both Systolic BP and BS, suggesting that these individuals maintain stable physiological parameters with minimal variation. Also, mid-risk cases (green) show significant overlap with both low-risk and high-risk cases, particularly in the range of systolic BP between 90 and 130 mmHg. This overlapping distribution suggests that mid-risk cases share physiological characteristics with both low- and high-risk groups, making classification inherently difficult. Thus, it can be seen that there is no clear decision boundary that separates mid-risk cases from other categories, reinforcing the challenge of accurately predicting this group. While high-risk cases generally exhibit more extreme values, mid-risk cases remain distributed throughout the range, likely contributing to lower Precision and Recall for mid-risk classification in the machine learning models.

In addition to machine learning methods, a conventional multinomial logistic regression analysis was conducted using SPSS, Version 29.0.2.0 (20) to assess the significance of individual physiological variables in predicting maternal risk levels. This statistical model allowed us to examine how each feature (e.g., Age, DiastolicBP, Blood Sugar) contributes to the likelihood of a case being classified as low-, medium-, or high-risk. The analysis revealed that not all variables were statistically significant predictors of maternal risk. For example, Age groups such as [Age = 10] and [Age = 14] returned non-significant *p*-values (Sig. > 0.95), indicating minimal predictive power. Conversely, variables like DiastolicBP = 70 (*p* = 0.034) and BS = 6.60 (*p* < 0.001) were found to be statistically significant in predicting a high risk, with odds ratios (Exp(B)) suggesting meaningful associations. These findings align with our machine learning results, where features such as Blood Sugar and Blood Pressure were also selected as relevant through data-driven feature selection (e.g., CfsSubsetEval in WEKA).

Although feature selection using the BestFirst + CfsSubsetEval method in WEKA consistently identified SystolicBP and Blood Sugar as the most informative predictors, the results from the multinomial logistic regression conducted in SPSS suggest that additional variables—such as DiastolicBP—may also hold significant predictive value, particularly in distinguishing the high-risk group. This variation is expected, as different methodologies capture distinct statistical relationships: machine learning-based feature selection often prioritizes interactions and redundancy, whereas regression focuses on individual variable significance in the presence of others. The convergence on SystolicBP and BS across both methods reinforces their clinical importance, while the broader results highlight the multidimensional nature of maternal risk prediction.

A comparison of the performances of the various methods proposed in the literature regarding the MHR classification problem and specifically the discrimination between high, medium, and low risk is presented at Table 10.

Alamsyah et al. [29] applied Random Forests with evolutionary weighting to the MHR dataset and obtained the best Accuracy of 82.18% using 10-fold cross-validation. Its findings reveal the potential of ensemble learning algorithms for maternal health risk prediction. However, our study addresses the class imbalance problem, which is a crucial factor affecting real-world classification performance. Without handling class distribution issues, models may achieve inflated performance metrics for the majority classes while underperforming in minority classes, particularly mid-risk cases. The nearest competitor to this work, Khadidos et al. [9], trained the MHR dataset using Gradient Boosted Trees. Its best Accuracy of 86% was achieved by using 10-fold cross validation. Its comparative better Accuracy with Random Forests proves that boosting helps improve model performance. Their results suggest that boosting techniques can further enhance model performance by sequentially correcting errors from previous iterations. However, their work lacks a comparative evaluation against other ensemble methods like Bagging or Adaptive Boosting, making it unclear whether Gradient Boosted Trees are indeed the optimal approach for this task. In this regard, another similar study is the work of Noviandy et al. [30], which used LightGBM on a comparable dataset and reported an Accuracy of 84.73%. Although these competitive Accuracy levels have been attained, their models result in underperformance. Raihen and Akter [31] attempted SVM with GridSearch optimization on the MHR dataset and obtained the highest Accuracy among all the traditional machine learning models, of 86.13%. Their experiment clearly showed that hyperparameter optimization is necessary for an improved SVM model at least in datasets where complex patterns are present. However, SVMs struggle with scalability when applied to larger datasets, and their reliance on kernel functions introduces additional computational overheads. Moreover, alternative optimization strategies, such as feature selection or cost-sensitive learning, which could further enhance classification performance could be included. Furthermore, Rahman et al. [14] applied SVM with an 80–20% training–testing split, achieving 79% Accuracy. The lower Accuracy compared to the other experiments may be due to a smaller-sized training set or not applying any kind of advanced preprocessing step like oversampling or feature selection. Also, Togunwa et al. [32] proposed a new MaternalNET-RF framework by using stratified K-fold cross-validation and obtained a very high 95% Accuracy. The very high performance of their model clearly demonstrated the strength of neural networks when combined with Random Forests for capturing complex patterns present in the data. Still, the high computational complexity of this hybrid model may be problematic in practical applications.

In comparison, the Accuracy obtained in this study was 88.03% with Random Forests implemented using 10-fold cross-validation. The contribution of SMOTE was remarkable, mainly by addressing class imbalance. Although the Accuracy is lower compared to that reported by Togunwa et al. [32], our approach provides the best computational efficiency, with good robustness, and hence is much more viable for practical applications in pregnant women’s risk prediction. The results have shown the great potency of Random Forests and the relevance of preprocessing in tackling imbalanced datasets. These findings confirm the state-of-the-art performance of both ensemble methods and deep learning models in previous works on maternal health risk prediction. Further research is needed to address major issues of class imbalance using more effective techniques like oversampling and to develop hybrid approaches that can pool the strengths from multiple algorithms. Multi-center validation and translation into clinical workflows remain to be performed in order to tap into the maximum potential of these machine learning tools to improve maternal health outcomes worldwide.

Many studies on maternal health risk prediction primarily focus on distinguishing high-risk from low-risk cases, as these are the most clinically significant and easier to differentiate. However, the mid-risk category remains largely overlooked, despite its crucial role in early intervention and preventive care. Our study addresses this gap by analyzing class overlap and enhancing classification performance for mid-risk cases. To achieve this, we employ SMOTE, an effective technique for rebalancing the dataset, ensuring that models receive sufficient training data to improve mid-risk predictions and mitigate biases introduced by class imbalance.

In fact, this study highlights the need for innovative strategies using machine learning-based approaches for the effective reduction in maternal health risks. While there is an improvement in healthcare, there is no doubt that maternal and infant mortality is one of the greatest challenges facing the world today [33]. In this context, machine learning offers a revolutionary opportunity through early identification and management of the maternal health risk, enabling more individualized and evidence-based care. Since machine learning algorithms, such as Random Forests and neural networks, can automatically process large volumes of data to uncover complex patterns, they are going to be very helpful in identifying a pregnancy at high risk and in guiding clinical decisions. These methods will enable healthcare providers to reduce preventable complications and reduce disparities in receiving quality care related to pregnancy [10].

Integrating machine learning into midwifery care, including predictive models for maternal health risks, offers holistic approaches in an effort to improve the health of women. AI-driven tools may also empower midwives and other healthcare professionals with specific interventions, even in resource-constrained or complex conditions that improve continuity and quality of care for mothers and infants [34,35]. This study also demonstrated that machine learning algorithms can classify risk accurately in maternal health, further reinforcing their potential as an essential part of modern midwifery care [36]. Such technologies embedded within maternal health systems allow for the creation of a more proactive and egalitarian data-driven approach toward better maternal and child health on a global scale.

The application of machine learning in Clinical Decision Support Systems (CDSSs) has the potential to significantly enhance maternal healthcare by providing data-driven risk assessments that assist clinicians in early intervention and treatment planning. CDSSs leverage patient data, including vital signs and historical health records, to generate automated risk predictions, supporting obstetricians, midwives, and other healthcare professionals in identifying high-risk pregnancies with greater Accuracy and efficiency [10]. By embedding such models within electronic health record (EHR) systems or mobile health platforms, healthcare providers can receive real-time alerts and personalized recommendations based on the patient’s risk level. This not only facilitates timely clinical decision-making but also contributes to reducing preventable maternal complications by enabling early intervention strategies [37].

Even though this study depicts the potential of machine learning algorithms in maternal health risk classification, there are few limitations that must be mentioned. First, the dataset used in this study was collected from a hospital in Bangladesh, which may introduce regional and demographic biases. While the dataset provides valuable insights, its applicability to other populations remains uncertain due to potential differences in maternal health risk factors, healthcare access, and socio-economic conditions across different countries and regions. Additionally, as demographic details such as ethnicity, socio-economic background, and clinical history are not provided in the dataset, the model’s performance outside of this specific setting requires further investigation. To enhance generalizability, future research should validate the model using multi-center and multi-population datasets, ensuring robustness across diverse healthcare systems. Furthermore, while this study uses a dataset of 1014 instances, this is relatively small for training complex machine learning models—especially neural networks—which may result in a poor generalization performance. Consequently, larger and more diverse datasets are of interest to enhance robustness and applicability to different populations.

Fewer representatives of the “mid risk” category definitely create class imbalance in the dataset and affect the performance of classifiers, as can clearly be seen from the small Precision and Recall values which belong to “mid risk”. The classes can either be balanced by over- or undersampling of the original data or by generating synthetic data with the use of algorithmic methods. However, the present study has concentrated on the technical performances of the algorithms and lacks socio-economic and contextual factors such as access to healthcare and social determinants of health—which are known to strongly influence maternal health outcomes—and thus limits any broad generalization of the results. It is only with such addition of data that future research can be conducted and improve the predictability of machine learning models further.

Finally, while there is a great promise from the machine learning models, how that promise will be translated into clinical practice remains the key challenge. Interpretability, usability in real-world healthcare, and the ethical implications of deploying an AI-based system in maternal health remain relevant concerns [38]. These points warrant deliberation in future studies so that the approaches proposed will translate into improved maternal and child health outcomes.

## 5. Conclusions

Maternal health risk prediction is among the most important research areas, which can greatly improve the health outcomes of mothers and their offspring. The ability to determine risk factors in advance and launch timely interventions will go a long way in decreasing maternal mortality and addressing complications during pregnancy and childbirth. Machine learning has been the game-changer in this field by analyzing complicated datasets, finding patterns, and making accurate predictions when traditional methods would otherwise fail. The integration of machine learning into maternal healthcare holds a number of advantages, such as speeding up decision-making processes, improving Accuracy in risk assessment, and raising the capability of handling vast and diverse data sources.

Healthcare providers may proactively use machine learning algorithms in maternal care by targeting interventions to the places and situations in which they are most needed. This will lead to improved short-term outcomes for both mothers and infants and have long-term health implications. In addition, as machine learning continues to develop, a key avenue of application will be in maternal health, to bridge the gaps in health access, decrease disparities, and contribute to achieving global health goals.

## Figures and Tables

**Table 1:** Distribution of instances across the risk classes.

	Low-Risk Class	Mid-Risk Class	High-Risk Class
# instances	406	336	272

**Table 2:** Descriptive statistics of the six physiological variables used in the classification.

	Count	Min.	Max.	Mean	STD	25%	50%	75%	Skewness	Kurtosis
Age	1014	10	70	29.87	13.47	19	26	39	0.783	−0.391
Systolic BP	1014	70	160	113.20	18.40	100	120	120	−0.251	−0.613
Diastolic BP	1014	49	100	76.46	13.89	65	80	90	−0.048	−0.949
Blood Sugar (BS)	1014	6	19	11.53	4.19	7	11	15	0.348	−1.244
Body Temperature	1014	98	103	98.67	1.37	98	98	98	1.747	1.436
Heart Rate	1014	7	90	74.30	8.09	70	76	80	−1.044	8.399

**Table 3:** Results of MHR classification for 10-fold cross-validation and the 6 classifiers in terms of Accuracy, TP Rate (average), and Precision (average). The bold values indicate the best performance across classifiers for each metric.

Metric	Naïve Bayes	Support Vector Machine	MLP	FCNN	Decision Tree	Random Forest
TP Rate	59.10%	75.70%	61.01%	68.91%	78.60%	**84.52%**
Precision	58.10%	76.30%	61.04%	68.96%	79.00%	**84.93%**
Accuracy	59.07%	75.74%	69.03%	68.93%	78.60%	**84.52%**

**Table 4:** Results of MHR classification for 80% training–20% testing split validation and the 6 classifiers in terms of Accuracy, TP Rate (average), and Precision (average).

Metric	Naïve Bayes	Support Vector Machine	MLP	FCNN	Decision Tree	Random Forest
TP Rate	59.60%	72.90%	64.50%	71.40%	76.80%	83.70%
Precision	57.40%	73.80%	64.60%	71.40%	76.20%	83.60%
Accuracy	59.61%	72.90%	64.53%	71.43%	76.85%	83.74%

**Table 5:** Confusion matrix for Random Forests, which showed the best classification Accuracy. The bold values indicate the best performance across classifiers for each metric.

			Predicted Values	
		High Risk	Low Risk	Mid Risk
Dataset	High risk	**90.07%**	3.31%	6.62%
	Low risk	2.22%	**81.03%**	16.75%
	Mid risk	5.65%	10.12%	**84.23%**

**Table 6:** Confusion matrix for Random Forests after model optimization.

Metric	TP Rate	Precision	Accuracy
Random Forests (SMOTE)	88.00%	88.10%	88.03%

**Table 7:** Confusion matrix for Random Forests after model optimization, which showed the best classification Accuracy. The bold values indicate the best performance across classifiers for each metric.

			Predicted Values	
		High Risk	Low Risk	Mid Risk
Dataset	High risk	**95.77%**	1.84%	2.39%
	Low risk	2.46%	**81.77%**	15.76%
	Mid risk	6.85%	10.12%	**83.04%**

**Table 8:** Results of confidence interval for the 6 classifiers in terms of Accuracy, TP Rate (average), and Precision (average).

Metric	Naïve Bayes	Support Vector Machine	MLP	FCNN	Decision Tree	Random Forest
TP Rate (95 CI)	58.44–60.07	67.81–75.34	61.33–70.81	67.31–72.49	77.00–82.18	83.62–87.76
Precision (95 CI)	51.20–59.16	66.00–73.50	62.00–68.44	65.12–74.11	75.23–80.09	78.12–85.15
Accuracy (95 CI)	58.44–60.07	67.81–75.32	61.33–70.80	67.32–72.48	77.01–82.19	83.62–87.76

**Table 9:** Paired t-test results comparing TP Rate, Precision, and Accuracy (mean and *p*-value) of various classifiers with Random Forests. Significant differences are indicated by *p*-values less than 0.05, with the corresponding 95% confidence intervals for the mean differences.

	TP Rate		Precision		Accuracy	
Model	Mean	*p*-Value	Mean	*p*-Value	Mean	*p*-Value
Naïve Bayes	26.113	0.000	26.046	0.000	26.118	0.000
Support Vector Machine	14.620	0.000	9.246	0.000	14.622	0.001
MLP	20.119	0.000	21.458	0.000	20.118	0.000
FCNN	13.322	0.000	13.225	0.000	20.104	0.000
Decision Tree	5.580	0.000	5.866	0.000	5.584	0.000

**Table 10:** Comparison of performances of the various methods proposed in the literature. The bold values highlight the performance metrics of the present study.

Study	Dataset	# of Cases	Evaluation Method	Classifier	Accuracy
Alamsyah et al. (2023) [27]	MHR dataset	1013	10-fold cross-validation	Random Forests and Evolutionary Weighting	82.18%
Khadidos et al. (2024) [9]	MHR dataset	1013	10-fold cross-validation	Gradient Boosted Trees	86.00%
Noviandy et al. (2023) [28]	MHR dataset	1014	10-fold cross-validation	LightGBM	84.73%
Raihen and Akter (2024) [29]	MHR dataset	1014	10-fold cross-validation	SVM (GridSearch)	86.13%
Rahman et al. (2023) [14]	MHR dataset	1013	80% training 20% testing	SVM	79.00%
Togunwa et al. (2024) [30]	MHR dataset	1014	Stratified K-fold cross-validation	MaternalNET-RF	95.00%
**This study**	**MHR** **dataset**	**1013**	**10-fold cross-validation**	**Random** **Forests**	**88.03%**

## Data Availability

No new data were created.
